# Identification and Functional Analysis of Known and New Mutations in the Transcription Factor KLF1 Linked with β-Thalassemia-like Phenotypes

**DOI:** 10.3390/biology12040510

**Published:** 2023-03-28

**Authors:** Rosa Catapano, Raffaele Sessa, Silvia Trombetti, Elena Cesaro, Filippo Russo, Paola Izzo, Alexandros Makis, Michela Grosso

**Affiliations:** 1 Department of Molecular Medicine and Medical Biotechnology, School of Medicine, University of Naples Federico II, 80131 Naples, Italy; 2 Department of Veterinary Medicine and Animal Productions, University of Naples Federico II, 80137 Naples, Italy; 3 Department of Pediatrics, University Hospital of Ioannina, Faculty of Medicine, School of Health Sciences, University of Ioannina, 45110 Ioannina, Greece

**Keywords:** KLF1, thalassemia, globin gene switching, gene expression, HbF, HbA_2_

## Abstract

**Simple Summary:**

A key regulator of erythropoiesis is the erythroid transcriptional factor Krüppel-like factor 1 (KLF1). Increased fetal hemoglobin (HbF) and hemoglobin A2 (HbA2) levels have been associated with KLF1 haploinsufficiency mutations and have been shown to mitigate the severity of β-thalassemia. In our study, two novel KLF1 haploinsufficiency mutations that result in enhanced fetal-globin gene expression, C94X and P173fxP236, are described and functionally characterized. Furthermore, our analysis also shows that some in cis KLF1 mutation combinations may aggravate the phenotype of β-thalassemia. Further understanding of the multiple functions of KLF1 in erythropoiesis is achieved by this study, which also emphasizes the possibility that a subset of KLF1 mutations may be responsible for the severity of the thalassemia phenotype. These findings support the relevance of KLF1 screening for genetic counseling and the efficacy of programs for hemoglobinopathies prevention screening.

**Abstract:**

The erythroid transcriptional factor Krüppel-like factor 1 (KLF1) is a master regulator of erythropoiesis. Mutations that cause KLF1 haploinsufficiency have been linked to increased fetal hemoglobin (HbF) and hemoglobin A_2_ (HbA_2_) levels with ameliorative effects on the severity of β-thalassemia. With the aim of determining if KLF1 gene variations might play a role in the modulation of β-thalassemia, in this study we screened 17 subjects showing a β-thalassemia-like phenotype with a slight or marked increase in HbA_2_ and HbF levels. Overall, seven KLF1 gene variants were identified, of which two were novel. Functional studies were performed in K562 cells to clarify the pathogenic significance of these mutations. Our study confirmed the ameliorative effect on the thalassemia phenotype for some of these variants but also raised the notion that certain mutations may have deteriorating effects by increasing KLF1 expression levels or enhancing its transcriptional activity. Our results indicate that functional studies are required to evaluate the possible effects of KLF1 mutations, particularly in the case of the co-existence of two or more mutations that could differently contribute to KLF1 expression or transcriptional activity and consequently to the thalassemia phenotype.

## 1. Introduction

Beta (β) thalassemia is a very heterogeneous disease ranging from asymptomatic conditions to severe anemia due to the effects of a large variety of ameliorating or worsening factors [1]. The major ameliorating factors associated with milder β-thalassemia phenotypes are the coinheritance of α-thalassemia mutations and the persistent expression of the normally repressed fetal hemoglobin (α_2_γ_2_, HbF) in adult life (hereditary persistence of fetal hemoglobin, HPFH). Therefore, interest has been growing regarding fetal globin gene reawakening as a novel therapeutic strategy for these disorders [2,3]. HPFH is commonly associated with large deletions affecting the β-globin gene cluster (deletional HPFH) or point mutations (non-deletional HPFH) in the promoter region of fetal globin genes (HBG1 and HBG2) indicating the presence of regulatory elements interacting with transcription factors that are potentially relevant to the fetal-to-adult globin switch [4,5,6,7,8].

**Figure 1:**
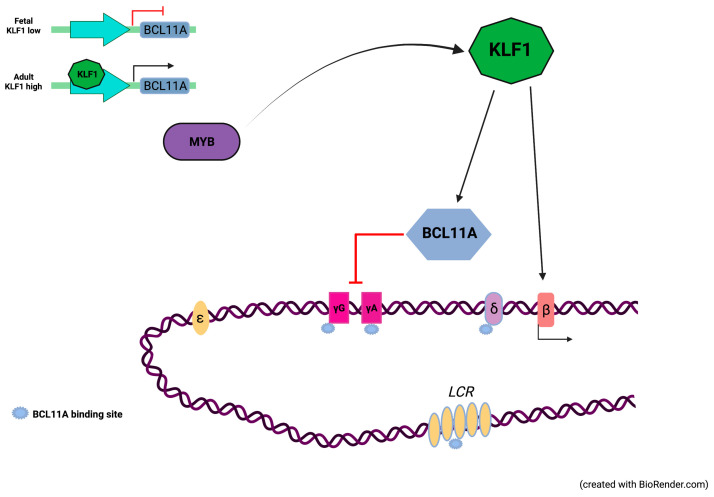
Globin gene activation is dependent upon the expression levels of KLF1. KLF1 directly activates the β-globin gene (HBB) and indirectly represses γ-globin genes (HBG1/2) through BCL11A, a repressor of γ-globin gene expression. In the fetal stage, limited KLF1 levels are associated with low expression levels of BCL11A, consequently fetal globin genes are expressed at high levels. In the adult stage, KLF1 is subject to transcriptional activation by c-Myb. At high levels, KLF1 promotes transcriptional activation of HBB and BCL11A thus contributing to the fetal-to-adult globin gene switching (Created with BioRender.com, accessed on 2 September 2021. Agreement number JR22WOKLFG).

In the last decades, several studies have contributed to identifying genetic factors located outside the β-globin locus, which are responsible for increased levels of HbF. Among these factors, KLF1 exerts a dual role in fetal-to-adult globin gene switching by directly activating the β-globin gene (HBB) and indirectly repressing fetal globin gene expression through the activation of BCL11A and ZBTB7A, a repressor of fetal globin gene (HBG1 and HBG2) expression (Figure 1) [9,10,11,12,13,14].

KLF1, formerly known as erythroid Krüppel-like factor (EKLF) for its restricted expression in erythroid cells and its similarity to the protein encoded by the Drosophila segmentation Krüppel gene, plays a multifunctional role in the regulation of a variety of cellular events leading to erythroid differentiation, including erythroid lineage commitment, heme synthesis, and globin gene switching [13,15,16,17]. The *KLF1* gene (~3 Kb) maps to chromosome 19p13.2 and consists of three exons encoding a 362-amino-acid protein with an *N*-terminal region rich in proline and containing two short transactivation domains (TAD1 and TAD2) and a *C*-terminus with three highly conserved Cys2/His2 zinc finger domains (ZF1, ZF2 and ZF3) that recognize the sequence motif 5′CCMCRCCCN3′ in CACCC boxes and GC-rich elements located within regulatory regions of its target genes [15]. 

Mutations in the *KLF1* gene have been reported to interfere with the erythropoiesis process, thus leading to severe hematological disorders, including congenital dyserythropoietic anemia (CDA) and congenital hemolytic anemia [18,19,20]. Additionally, KLF1 haploinsufficiency contributes to a number of benign hematological conditions, such as borderline-elevated HbA_2_ levels, mild microcytosis, and/or hypochromia [21,22]. Importantly, monoallelic KLF1 mutations have also been reported to be associated with increased levels of HbF, thus contributing to ameliorate the clinical severity of β-thalassemia [22,23,24,25,26]. However, although in the last years the contribution of KLF1 in human erythropoiesis has been disclosed, there are still unanswered questions that need to be addressed to better clarify the significance of different KLF1 mutations on these phenotypes.

Here, we report the identification and functional characterization of known and novel mutations in the promoter and coding regions of KLF1. In this study, we also examined the potential synergistic effect of combined mutations over single point mutations.

Given the potential therapeutic role of HPFH in hemoglobinopathies, this study sheds new information on the role of KLF1 in erythropoiesis and draws attention to the notion that certain KLF1 genotypes may contribute to enhance KLF1 activity thus acting as worsening factors in the thalassemia phenotype.

## 2. Materials and Methods

### 2.1. Patients and Hematological Data

This study was performed on 17 subjects showing a β-thalassemia-like phenotype including 9 β-thalassemia heterozygotes with mild/severe microcytic anemia, elevated HbA_2_ and/or HbF levels and 8 subjects with normal or borderline erythrocyte indices, normal or borderline HbA_2_, HbF levels between 0.8 and 6.6%, with normal α- and β-globin gene genotypes, and normal serum iron values. Hematological parameters were determined using an automatic cell counter (ADVIA 2120 I, Siemens, Munich, Germany) and high-performance liquid chromatography (Variant II, Bio-Rad Laboratories Inc., Hercules, CA, USA). The study design was approved by our local Institutional Research Ethics Committee (project approval number 443/21). The study was conducted according to the criteria set by the Declaration of Helsinki and the Belmont Report. Informed consent was obtained from individuals or their guardians for all samples used in this study.

### 2.2. Genetic Analysis of HBA and HBB Clusters

Genomic DNA was extracted from peripheral blood using the Nucleon BACC3 kit (GE Healthcare, Life Sciences, Chicago, IL, USA). 

Screening for mutations in the HBA and HBB clusters was carried out by Sanger sequencing on an automated DNA sequencer (ABI PRISMTM 3130 XL, Life Technologies, Carlsbad, CA, USA), amplification refractory mutation system (ARMS), and Multiplex-Ligation-dependent Probe Amplification (MLPA P102-C1 HBB probe mix and P140-C1 HBA probe mix) (MRC-Holland, Amsterdam, The Netherlands) as previously reported [27,28,29,30,31].

### 2.3. Cell Culture

The human K562 myeloid leukemia cell line provided by the European Collection of Authenticated Cell Cultures (EACC #89122407) was maintained in RPMI 1640 medium supplemented with 10% fetal bovine serum (FBS) plus 4 mM glutamine (all reagents from Gibco, Thermo Fisher Scientific Inc., Waltham, MA, USA) at 37 °C in a humidified 5% CO_2_-containing atmosphere. The cells were kept sub-confluent for transient transfection experiments [32].

#### 2.3.1. Transient Transfection

K562 cells were transiently transfected with a mix containing 1 μg of p3XFLAG-KLF1 wild-type, 1 μg of each mutant constructs, 1 μg of p3XFLAG-CMV empty vector (mock control), and 5 μL of Lipofectamine 2000 as the transfection reagent (Invitrogen, Carlsbad, CA, USA). Two hours before transfection, the cells were plated into six-well plates at a density of 4 × 10^5^ cells/mL in 2 mL of RPMI 1640 without the addition of serum and antibiotics.

Five hours after transfection, the medium was supplemented with 10% FBS in each well. The plates were incubated in the appropriate growth conditions (37 °C, 5% CO_2_) for 48 h. The cells were harvested for RNA and protein extraction.

#### 2.3.2. RNA Extraction

Total RNA was extracted from K562 cells with QIAzol reagent (Qiagen GmbH, Hilden, Germany) according to the manufacturer’s protocol. After spectrophotometric quantization, RNA quality was checked by gel electrophoresis on a 1.5% denaturing agarose gel in MOPS 1× buffer (20 mM MOPS pH 7.0, 8 mM sodium acetate, and 1 mM EDTA pH 8.0). 

#### 2.3.3. Protein Extraction

For protein extraction, K562 cells were collected 48 h after transfection and washed twice with 3 to 4 mL of cold 1× PBS by centrifugation at 3000× *g* for 10 min at 4 °C. The pellets were resuspended in 100 μL of lysis buffer (10% glycerol, 50 mM Tris-HCl pH 8.0, 150 mM NaCl, 0.1% NP-40, 1 mM EDTA pH 8.0, and 10 μL of protein inhibitor cocktail mixture (Sigma-Aldrich, St. Louis, MO, USA)) and incubated for 30 min on ice. The samples were then centrifuged at 10,000× *g* for 30 min at 4 °C and the supernatant containing total protein extract was collected. Evaluation of protein concentration was performed as previously reported [33].

### 2.4. Generation of KLF1 Mutants by Site-Specific Mutagenesis

Amplifications of KLF1 full-length cDNA and the promoter region were performed using the primers listed in Table 1. The cDNA was cloned in the expression vector p3XFLAG-CMV10, whereas the promoter fragment was cloned in the pGL4.10 [luc2] luciferase gene reporter vector.

Point mutations in the wild-type constructs were introduced by PCR, using the QuickChange II XL Site-Directed Mutagenesis kit (Agilent Technologies, Santa Clara, CA, USA) according to the manufacturer’s instructions. Site-directed mutagenesis reactions containing 5 μL of 10× reaction buffer, 1 μL of 10 ng/μL template, 10 pmol of each primer, 1 μL of dNTP mix, 3 μL of Quick solution, H_2_O to 50 μL, and 1 μL (2.5 U) PfuUltra HF polymerase were run on a MyCycler thermal cycler (Bio-Rad Laboratories, Hercules, CA, USA). 

PCR reactions were run with an initial denaturation step at 95 °C for 2 min followed by 18 cycles at 95 °C for 1 min, 60 °C for 50 s and 68 °C for 15 min, and a final elongation step at 68 °C for 7 min. After amplification, 1 μL of the Dpn I restriction enzyme (10 U/μL) was added to each sample. The reaction was incubated at 37 °C for 90 min.

The mutant constructs (KLF1-S102P, KLF1-F182L, KLF1-M39L, KLF1-C94X KLF1-P173PfsX236, KLF1-S102P/F182L, KLF1-M39L/S102P, KLF1-251, KLF1-148, and -251/-148) were transformed into XL-10 Gold cells and successful mutagenesis was confirmed by DNA sequencing.

### 2.5. Real-Time PCR Analysis

To determine the expression levels of KLF1 mutants and its target genes, namely HBB, BCL11A, ZBTB7A, and the fetal globin genes HBG1/HBG2, quantitative real-time PCR analysis was performed on a CFX96 real-time system (Bio-Rad Laboratories, Hercules, CA, USA). cDNA was synthesized from 500 ng of total RNA extracted from K562 cells, using the QuantiTect Reverse Transcription kit (Qiagen, Hilden, Germany). Each reaction was performed according to the procedure recommended by the manufacturer and subsequently used for PCR analysis.

Each real-time PCR was performed in triplicate in a 20 μL reaction mix containing 10 μL of 2× SsoAdvanced Universal SYBR Green Supermix (Bio-Rad Laboratories, Hercules, CA, USA), 0.38 μL of a 20 μM primer mix, 2 μL of cDNA (1:100 of RT-PCR product), and 7.62 μL of nuclease-free water. According to GenBank sequences, primers for all transcripts were designed for quantitative real-time PCR analysis. β2-microglobulin mRNA was used as a reference control. Primers used for KLF1 and ZBTB7A were as previously reported [34,35]. All primer sequences are listed in Table 2.

Cycling conditions consisted of an initial denaturation step at 95 °C for 3 min, followed by 40 cycles (95 °C for 15 s, 60 °C for 30 s). Melting curve analysis of amplicons was performed according to standard protocols. The calibration curve for assessing the efficiency of the PCR reaction was performed on at least three serial dilutions (1:10) of the reverse transcriptase products. CT values were determined by automated threshold analysis and data were analyzed by the CFX Manager 3.0 software (Bio-Rad Laboratories, Hercules, CA, USA) according to the manufacturer’s specifications. Untransfected K562 cells and/or K562 cells transfected with empty vectors were used as mock controls.

### 2.6. Western Blot Analysis

Western blot analysis was performed on 30 µg of total protein extracts according to the protocol previously described. Proteins were resolved on 10% SDS-page gels and transferred to nitrocellulose membranes by the Trans-blot Turbo instrument (Bio-Rad, Hercules, CA, USA). After blotting, the filters were blocked and then hybridized at 4 °C for 2 h with anti-FLAG antibody (1:5000 dilution; Sigma-Aldrich, Saint Louis, MO, USA). The membranes were washed thrice with 1× TBS-Tween 20 buffer for 5 min and incubated for 45 min with a peroxidase-conjugated anti-mouse IgG secondary antibody (1:10,000 dilution, Bio-Rad Laboratories, Hercules, CA, USA). The antigen–antibody complexes were then detected using the ECL Immobilon Western Chemiluminescent HRP-substrate system (Millipore, Darmstadt, Germany) and autoradiography, according to the manufacturer’s instructions. Signals were subsequently normalized with an antibody anti α-actin (dilution 1:10,000; Sigma-Aldrich, Saint Louis, MO, USA). Western blots bands were quantified using ImageJ software 1.53a (Bio-Rad Laboratories, Hercules, CA, USA).

### 2.7. Luciferase Gene Reporter Assay

K562 cells were transfected using Lipofectamine 2000 (Thermo Fisher Scientific, Waltham, NJ, USA). Two hours before transfection, the cells were resuspended in Optimem without serum and antibiotics, at a density of 2 × 10^5^ cells/mL. The cells were then seeded in 24 multiwell plates in a final volume of 500 μL of RPMI. For each experiment, the cells were transfected in triplicate with the following constructs: pGL4luc-PromKLF1WT (wild-type), pGL4luc-PromKLF1–251, pGL4 luc-Prom KLF1–148, pGL4luc-PromKLF1–251/−148, and PGL4null (negative control). In addition, gene reporter assays were performed by co-transfecting K562 cells with a vector (pGL4luc-Promβ) containing the β-globin proximal promoter region from −163 to +37 that comprises a KFL1 binding site located between −90 to −105 [36], and the following expression vectors: p3XFLAG wild-type, p3XFLAG-S102P, p3XFLAG-F182L, p3XFLAG-S102P + F182L, p3XFLAG-M39L, p3XFLAG-M39L + S102P, p3XFLAG-P173fsX236, p3XFLAG-C94X and p3XFLAGXNull. To normalize luciferase assays, 25 ng of pRL-CMV vector (Promega, Madison, WI, USA) coding for Renilla were co-transfected with 225 ng of pGL4 luc constructs. For each sample, the cells were transfected with 250 ng of DNA mix (pGL4 luc/pRL-CMVRenilla) in Optimem culture medium (GIBCO) without serum up to 100 μL of the final volume and 1.5 Lipofectamine 2000. The solution was gently stirred, incubated for 15 min at room temperature and then distributed directly to the cells that were incubated for 48 h, according to the standard growth conditions (37 °C, 5% CO_2_). Forty-eight hours after transfection, the luciferase assay was performed using the Dual-Luciferase Reporter Assay System kit (Promega, Madison, WI, USA) as previously reported [37].

### 2.8. Statistical Analysis

All data were assessed as the mean ± standard deviation (SD) of at least three separate experiments performed in triplicate. GraphPad Prism 7 (GraphPad Software, Inc., San Diego, CA, USA) was used for data analysis. Statistical differences were determined through the one-way analysis of variance procedure followed by Dunnett’s multiple comparison test, comparing results between the mock control, and treated cells. Differences were statistically significant when *p* < 0.05 (*) (#) and highly significant when *p* < 0.0001 (**) (##) versus each respective mock or KLF1 wild-type control group.

## 3. Results

### 3.1. Identification of Mutations in the Coding Region of KLF1 Gene and Production of Mutant Constructs

**Figure 2:**
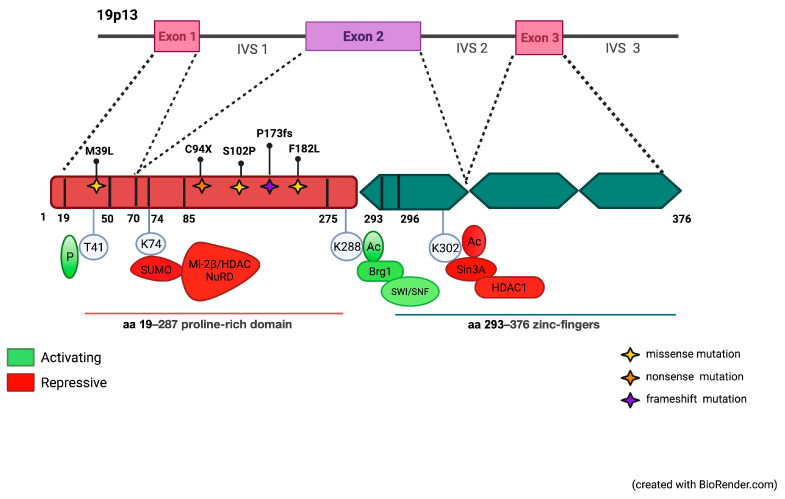
Schematic representation of the KLF1 gene and its protein domains. KLF1 activity is modulated by post-translational modifications promoting protein–protein interactions. Sites of phosphorylation, sumoylation, acetylation, and ubiquitinylation modifications and their effects on subsequent protein–protein interactions and transcriptional readouts are shown. Modifications that are essential for activation are shown in green; repressive modifications in red. Mutations in the proline-rich domain reported in this study are shown (Created with BioRender.com, accessed on 2 September 2021. Agreement number HD22WOKT15).

**Figure 3:**
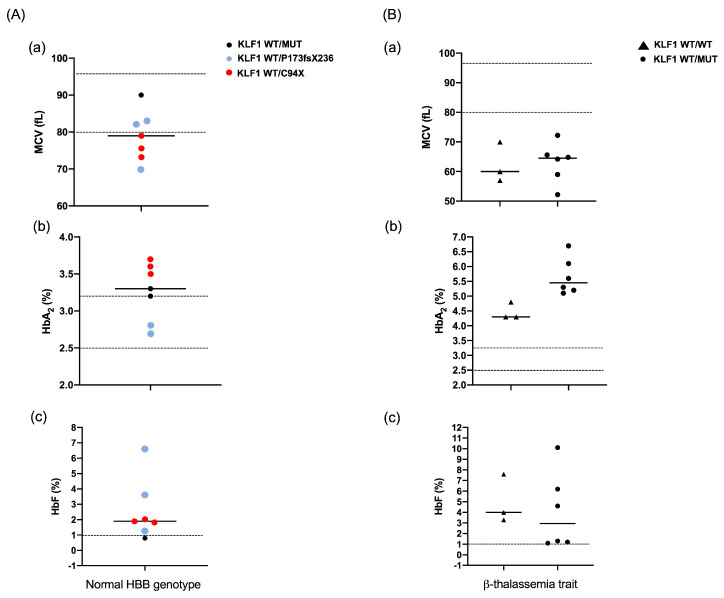
Effects of KLF1 mutations on hematological parameters. (**A**) MCV (**A**(**a**)), HbA_2_ (**A**(**b**)), and HbF (**A**(**c**)) levels in subjects heterozygous for KLF1 mutations with a normal HBB genotype. Red and blue circles correspond to values of subjects heterozygous for C94X and P173fsX236 mutations, respectively. Black circles correspond to heterozygotes for other pathogenic KLF1 variants; (**B**) MCV (**B**(**a**)), HbA_2_ (**B**(**b**)), and HbF (**B**(**c**)) levels in subjects with a β-thalassemia trait and wild-type (triangle) or mutant KLF1 (circle). For each parameter, horizontal lines indicate mean values. Reference range values are shown as dotted lines: MCV, 80–97 fL; HbA_2_, 2.5–3.2%; HbF, <1%.

Initially we performed a screening for the presence of mutations at the HBA1, HBA2 (α-globin) and HBB (β-globin) genes in subjects with mild or silent β-thalassemia phenotypes. Nine subjects resulted to be carriers of one or more mutations in the β-globin gene with an αα/αα genotype, whereas eight subjects showed normal α- and β-globin genotypes which were not consistent with altered red blood cell indices, including low mean corpuscular volume (MCV) and elevated HbA_2_ and/or HbF levels (Table 3). We then proceeded to screen all subjects for variations in the KLF1 gene. Overall, we identified five different variations in the KLF1 coding region, two of them being yet unreported mutations in thirteen individuals, including six β-thalassemia carriers and seven subjects with normal α- and β-globin genes (Table 3 and Figure 2). The novel variants were a nonsense mutation (C94X) and a frameshift mutation (P173fsX236), both found in the transacting proline-rich domain of exon 2. C94X creates a premature stop codon at codon 94 (TGC > TGA) and was detected at the heterozygous state in three related individuals, all of them with slightly increased HbF levels (1.8–2.0%), borderline elevated HbA_2_ levels (3.5–3.7%), and normal α- and β-globin genotypes (Table 3, subjects 10, 11 and 14). DNA sequencing of all family members revealed the presence of this mutation only in the individuals showing increased HbF and HbA_2_ levels. The other novel mutation, the P173fsX236 frameshift, produces a premature stop codon at position 236 and was identified in two unrelated subjects, both with normal α- and β-globin gene genotypes (Table 3, subjects 13 and 16). In one case, the mutation was detected in a subject with 1.3% HbF and borderline HbA_2_ levels (3.3%) (Table 3, patient 13); in the other case, it was found in association with the S102P and F182L variations, in a subject with 6.6% HbF and normal levels of HbA_2_ (Table 3, patient 16). Among known variations, p.S102P (c.304T > C), p.F182L (c.544T > C), and p.M39L (c.115A > C) variations were detected. S102P, which falls in the proline-rich domain and involves the substitution of a serine for proline at codon 102 (TCC > CCC) is the most-frequent variation found in our study, either alone or in association with other mutations. It had been previously reported as an unlikely pathogenic SNP (rs2072597) [38]. The p.F182L variation is also classified as a benign variant (rs2072596) falling in the proline-rich domain and involving a phenylalanine-for-leucine substitution at codon 182 (TTC > CTC) [39,40]. In our study, it has been found in combination with other KLF1 sequence variations in a β-thalassemia heterozygous (Table 3, subjects 3) and in two subjects with normal α- and β-globin genotypes. The p.M39L (c.115A > C) variation falls in the transactivation domain encoded by exon 1 and involves a methionine for leucine substitution at codon 39 (ATG > CTG) [41]. It is currently classified as probably benign (rs112631212) and, in our study, it has been found in cis with S102P in two related subjects both heterozygous for a β-thalassemia mutation (Table 3, subjects 12 and 15). Lower MCV values, along with increased HbA_2_ levels were observed in KLF1 variant-positive subjects with normal α- and β-globin genotypes whereas the presence of KLF1 variants significantly contributed to increasing HbA_2_ levels in β-thalassemia carriers (Figure 3).

### 3.2. Functional Characterization of KLF1 Variants

**Figure 4:**
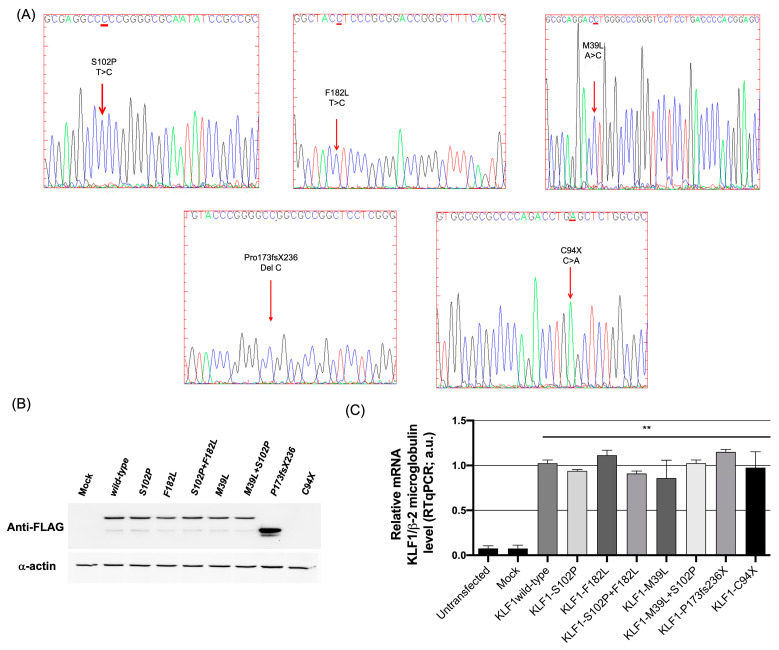
Evaluation of expression levels of KLF1 mutants in K562 cells. (**A**) Sanger electropherograms showing the mutations generated in the KLF1 expression vector (p3XFLAG wild-type, p3XFLAG-C94X, p3XFLAG-P173fsX236, p3XFLAG-S102P, p3XFLAG-F182L, and p3XFLAG1-M39L); (**B**) Western blot analysis showing expression levels of normal and mutated KLF1 constructs in K562 cells transiently transfected with p3XFLAG wild-type, p3XFLAG-S102P, p3XFLAG-F182L, p3XFLAG-S102P + F182L, p3XFLAG-M39L, p3XFLAG-M39L + S102P, p3XFLAG-P173fsX236, and p3XFLAG-C94X) (Figure S1 shows the original image in the supplementary material); (**C**) Real-time PCR analysis showing expression levels of endogenous KLF1 in untransfected K562 cells and in K562 cells transiently transfected with wild-type and mutant KLF1 vectors or the mock control. All data represent the mean ± SD of three independent experiments. Statistical analysis was performed by one-way ANOVA, followed by Dunnett’s multiple comparisons test. Differences versus untransfected cells and mock controls were considered highly significant when *p* < 0.0001 (**). ANOVA: analysis of variance; SD: standard deviation.

To investigate the causative effect of the missense or nonsense variants detected in KLF1, we performed functional studies in K562 cells. To this aim, wild-type and mutant KLF1 cDNAs were cloned into the p3XFLAG-CMV plasmid expression vector. Mutant vectors were obtained by site-directed mutagenesis reactions. Additionally, in some cases, families studied allowed us to assess a monoallelic inheritance of two KLF1 variations (Table 3). Therefore, to evaluate the combined effects of these variants, mutant recombinant vectors corresponding to these genotypes were generated by introducing the F182L and M39L mutations, respectively, in the construct bearing the S102P substitution. DNA sequencing, Western blotting and quantitative real-time PCR analysis were performed to check the presence of selected mutations in these constructs and to verify their expression levels indicative of similar transfection efficiency of the constructs (Figure 4). As expected, a signal corresponding to a truncated protein was observed in the Western blot corresponding to the frameshift mutation P173fsX236 whereas no signals were detected for the nonsense mutation C94X due to the premature amino acid termination position of this variant that, differently from the frameshift mutation which is located closer to the 3′ end of the transcript, is predicted to elicit a mechanism of nonsense-mediated mRNA decay (NMD) that abolish the synthesis of abnormal truncated proteins by triggering mRNAs degradation [42]. In any case, by quantitative real-time PCR analysis, we found that the mRNA levels of the C94X construct were comparable to those of other constructs (Figure 4C), thus indicating that pathways alternative to NMD could be involved in the lack of protein product for this construct. Indeed, it has been shown that Upf1, a key NMD factor, triggers a proteasome-mediated degradation of aberrant proteins originated from mRNAs with a premature stop codon, as an alternative mechanism to avoid the production of potentially dangerous protein products [43,44]. Therefore, based on our findings, we speculate that such a mechanism involving a proteasome-mediated Upf1 pathway may explain the degradation of the aberrant C94X protein without inducing mRNA decay.

**Figure 5:**
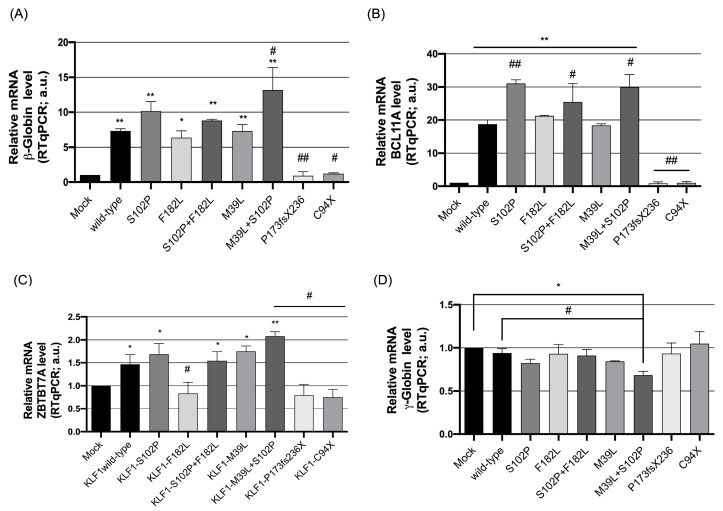
Expression levels of KLF1 target genes in K562 cells transiently transfected with wild-type and mutant KLF1 vectors. Real-time PCR analysis of (**A**) β-globin (HBB), (**B**) BCL11A, (**C**) ZBTB7A, and (**D**) γ-globin (HBG1/2) mRNA levels in K562 cells transfected with wild-type and mutant KLF1 constructs. β_2_-microglobulin was used as a reference gene for the relative normalization of gene expression analysis. Data are presented as fold-changes to the mock control. All data represent the mean ± SD of three independent experiments. Statistical analysis was performed by one-way ANOVA, followed by Dunnett’s multiple comparisons test, where appropriate. Differences were considered significant when *p* < 0.05 (*) (#) and highly significant when *p* < 0.0001 (**) (##) versus each respective mock and KLF1 wild-type control group. ANOVA: analysis of variance; SD: standard deviation.

To evaluate the significance of the KLF1 variants detected in our study, functional assays were performed in K562 cells transiently transfected with KLF1 wild-type or mutant expression vectors (S102P, F182L, S102P + F182L, M39L, M39L + S102P, P173Pfs236X and C94X constructs). The transcriptional activity of KLF1 was quantitatively determined by analyzing the mRNA levels of KLF1 target genes (Figure 5). We thus examined the effects of these constructs on the expression levels of HBB, BCL11A, ZBTB7A, and HBG1/2 according to the role of KLF1 in promoting hemoglobin fetal-adult switching by directly up-regulating HBB and indirectly repressing fetal globin gene (HBG1/2) expression through the up-regulation of the transcription factor BCL11A and ZBTB7A [13,14,45].

In relation to HBB, we found increased gene expression levels in K562 cells transfected with KLF1 wild-type; further increased β-globin transcriptional up-regulation was observed in the construct bearing the S102P variation whereas F182L and M39L mutants showed expression levels for β-globin that were comparable or only slightly reduced with respect to wild-type KLF1. Conversely, constructs bearing the S102P variant along with F182L or M39L showed a significant increase in HBB transcriptional levels, more statistically marked in cells expressing the M39L + S102P double mutant, thus suggesting that monoallelic inheritance of likely benign sequence variations may have potentiated synergistic effects on the transcriptional activity of KLF1. On the other hand, the two novel mutations identified in our study, P173Pfs236X and C94X, both haploinsufficiency mutations, were associated with a dramatic reduction in HBB expression levels (Figure 5A).

We also determined the levels of the BCL11A, ZBTB7A, and γ-globin transcripts. As expected, due to the embryo-fetal hemoglobin pattern of the K562 cell line, we found very low background levels of BCL11A and ZBTB7A accompanied by elevated HBG1/2 expression levels (mock controls, Figure 5B–D). In cells overexpressing wild-type and KLF1 variants, we also found a general inverse correlation between variations in BCL11A, ZBTB7A, and γ-globin gene expression levels for all the constructs in examination. Interestingly, cells transfected with the M39L + S102P double mutant construct showed a significant increase in the expression levels of BCL11A and ZBTB7A accompanied by a consistent decrease in γ-globin gene expression as compared to M39L single mutant and wild-type constructs (Figure 5B–D). These results further support the evidence that S102P enhances transcriptional activity with respect to M39L single mutant and wild-type constructs.

In K562 cells expressing the P173PfsX236 and C94X mutants, a dramatic reduction in the transcriptional activation of KLF1 target genes was observed as compared to the mock control, consistent with the haploinsufficiency effect predicted for these two variants [14,46]. This condition was accompanied by a slight increase in γ-globin gene expression levels that is to be considered of relevance given the presence of elevated background levels of fetal globin gene expression in K562 cells that may mask the effects of additional transactivation factors.

### 3.3. Evaluation of the Transcriptional Activity of KLF1 Variants on HBB Expression

**Figure 6:**
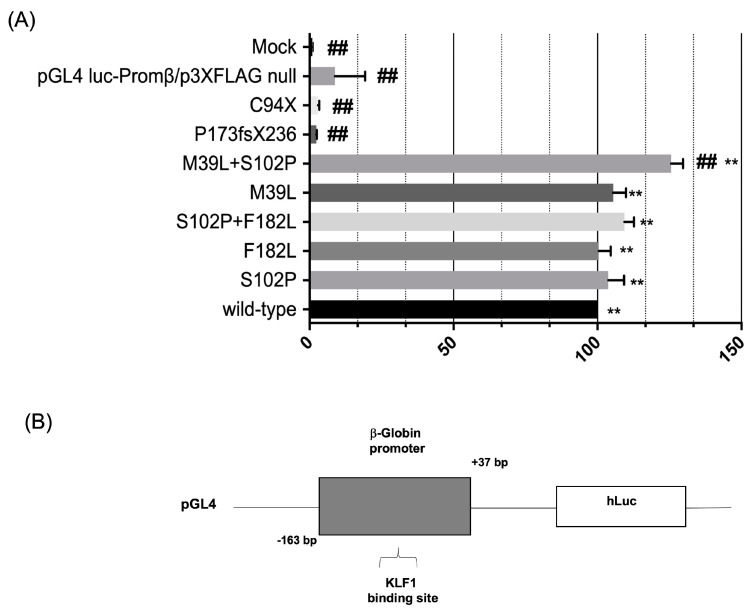
Effects of KLF1 mutations on β-globin production. (**A**) Luciferase activity assay in K562 cells co-transfected with a plasmid containing the β-globin promoter cloned 5′ to the luciferase reporter gene and KLF1 wild-type or mutant expression vectors. The histogram represents the relative firefly luciferase activity relative to the Renilla luciferase activity (relative luminescence units-LRU) expressed as percentage of activity of the β-globin promoter; (**B**) schematic representation of the luciferase gene reporter vector showing the β-globin promoter region containing a KLF1 binding site and cloned upstream the luciferase gene. All data represent the mean ± SD of three independent experiments. Statistical analysis was performed by one-way ANOVA, followed by Dunnett’s multiple comparisons test, where appropriate. Differences were considered highly significant when *p* < 0.0001 (**) (##) versus each respective mock or KLF1 wild-type control group. ANOVA: analysis of variance; SD: standard deviation.

To better dissect the transcriptional activity of these KLF1 variants, we examined in more detail the effects of wild-type and mutated constructs on the expression levels of HBB. To this aim, gene reporter assays with β-globin gene promoter driving the expression of firefly luciferase confirmed that expression variations of target genes are directly dependent on different transcriptional activity of KLF1 variants. Notably, a statistically significant variation was found for the M39L + S102P double mutant thus further confirming the synergistic effects of this variant composition. In addition, as expected, the expression of mutants generating truncated proteins, C94X and P173fxP236, was accompanied by a dramatically reduced transcriptional activity of the β-globin gene promoter compared to the mock control (Figure 6).

### 3.4. Identification and Functional Characterization of Sequence Variations in the KLF1 Promoter Region

**Figure 7:**
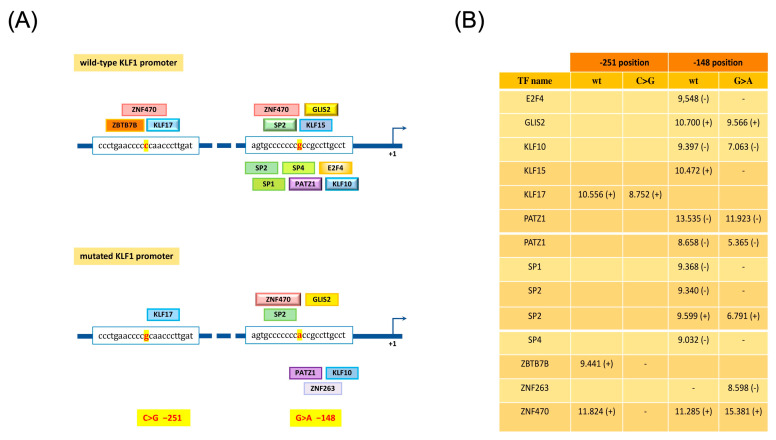
Prediction of transcription binding sites on wild-type and mutated KFL1 promoter sequences. (**A**) Prediction of transcription factors binding sites (TFBS) on KFL1 promoter, by JASPAR tool (score cutoff set at 8.5), reveals different transcription factor binding profiles on wild-type and mutated −251 C > G and −148 G > A sequences. (**B**) The table reports the significant TFBS scores found within forward (+) and reverse (−) strands.

In the course of our study, we also identified two known sequence variations in the promoter region, c. −251 (C > G) (rs3817621) and c. −148 (G > A) (rs79334031), reported as the benign polymorphism (SNP) and pathogenetic variant, respectively [47,48,49,50]. In our study, these two variations were found in cis in a 73-year-old man who was also heterozygous for the P173fxP236 variant in the KLF1 gene. This condition was associated with mild hypochromic microcytic anemia, 3.6% HbF and 2.8% HbA_2_ with normal α- and β-globin gene genotypes (Table 3, subject 16). The c. −251 (C > G) variation alone was also found in association with the S102P polymorphism in a 28-year-old woman with moderate anemia who was heterozygous for the β^0^39 mutation (Table 3, subject 4) and in association with the F182L mutation in a 30-year-old man negative for thalassemia mutations with borderline HbA_2_ levels (3.2%) (Table 3, patient 11). Furthermore, it was identified in trans to c. −148 (G > A) in patient five, who was also a carrier of the S102P variant and a β^0^-thalassemia mutation (Table 3). We employed GeneCards and JASPAR databases [51] for the search of putative transcriptional binding sites in these two regions. Bioinformatics analysis predicted three transcriptional factors that could bind the promoter region around position −251 (KLF17, ZBTZ7B and ZNF470). Significantly, the C > G substitution at position −251 was found to abolish two of these sites (ZBTZ7B and ZNF470). On the other hand, a higher number of transcriptional factors were predicted to bind the region around position −148 with the G > A substitution resulting in the disruption or activation of some of these transcriptional binding sites, including an abolished SP1 binding site as already reported [15,25,34,39] (Figure 7).

**Figure 8:**
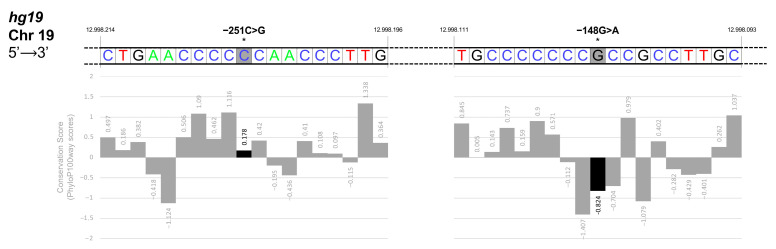
Base conservation scores in the KLF1 promoter region. In this figure, we represented the Conservation Scores of Chromosome-19 bases on the negative strand, showing the two segments containing mutation –251C and −148G positions (starred indicated position 12998205 and position 12998102, colored in grey, respectively). Below each base (colored-coded with blue for Cs, red for Ts, black for Gs, and green for As), we reported the respective PhyloP100way score from the VarSome database, presented in the form of a diagram. Based on 99 vertebrate genome sequences aligned to the human genome, the PhyloP100way score represents the individual alignment site conservation state. A positive score indicates sites that are predicted to be conserved; on the other hand, a negative score indicates sites that are predicted to be fast evolving. As shown in the lower panel (black bar graph), the slightly positive score for −251C (+0.178) indicates that this base position is poorly conserved whereas the intermediate value of the negative score for −148G (−0.824) is consistent with a moderately evolving base position.

To further characterize these promoter sites, we performed an analysis of base conservation scores for these two KLF1 promoter regions on 99 vertebrate genome sequences aligned to the human genome (represented by PhyloP100way scores provided by the VarSome platform, https://varsome.com/about/resources/acmg-implementation (URL accessed on 20 December 2022)). The slightly positive score for −251C (+0.178) corresponds to a poorly conserved base position whereas the intermediate value of the negative score for −148G (−0.824) is consistent with a moderately evolving base position. These findings further support a more significant functional role for −148G with respect to −251C and with results from a prediction analysis of putative regulatory elements in these two regions (Figure 8).

**Figure 9:**
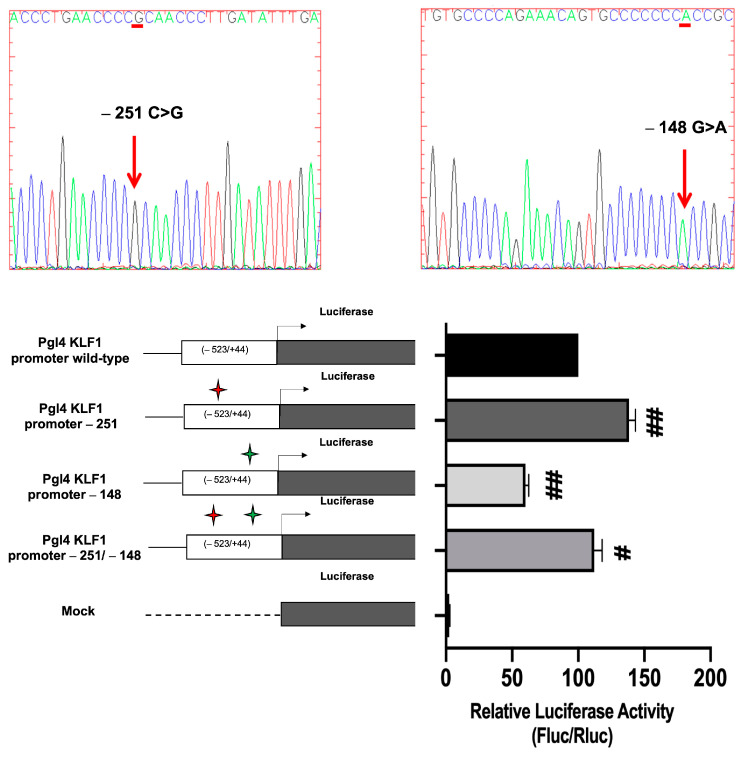
Luciferase activity assay in K562 cells transfected with mutants and wild-type constructs of the KLF1 promoter. (**Upper part**) Sanger DNA sequencing analysis showing the mutations generated in the KLF1 expression vector; (**lower part**) the histogram shows the mean values of Firefly and Renilla luciferase activity ratios (relative bioluminescence units-LRU) for each sample. Red and green stars indicate the presence of the −251 C > G and −148 G > A sequence variations, respectively, in the constructs in exam. The data shown are the mean ± SD of three independent experiments. Statistical analysis was performed by one-way ANOVA, followed by Dunnett’s multiple comparison tests, where appropriate. Differences were considered significant when *p* < 0.05 (#) and highly significant when *p* < 0.0001 (##) versus pGL4 KLF1 promoter wild-type.

To investigate the functional effects of these two promoter variants, K562 cells were transiently transfected with reporter constructs containing the firefly luciferase gene under the control of the wild-type or mutated KLF1 promoter region spanning from positions −523 to +44, relative to the transcriptional initiation site. Gene reporter assays indicated that, as compared to the wild-type construct, the c. −148 G > A mutation determines a significant reduction in the transcriptional activity of the KLF1 promoter, as expected on the basis of literature data [16,38,47]. Conversely, in cells transfected with the −251 pGL4luc-PromKLF1 construct, the luciferase activity was found to be 40% increased with respect to the wild-type construct. This was a rather unexpected finding, given the benign significance reported for this variant and the lack of putative regulatory elements identified in this promoter region. Collectively, our data raised the notion that this sequence variation is not to be considered to be a neutral polymorphism. More intriguingly, the construct bearing both sequence variations showed an increased transactivation activity when compared to the wild-type vector, although at a lower level with respect to the−251 pGL4luc-Prom KLF1 promoter construct. Therefore, these findings indicate that co-inheritance in cis of c. −148 (G > A) only partially reduced the transcriptional activity promoted by the −251 (C > G) variation. Consequently, besides confirming the opposite effects of these two sequence variations on the transcriptional activity of the KLF1 promoter, these results indicate that c. −251 (C > G) has a partially dominant effect on c. −148 (G > A) as it almost completely reverses its negative effects on KLF1 transcription (Figure 9).

## 4. Discussion

KLF1 gene variations are linked to a variety of hematological phenotypes ranging from severe hemolytic anemia to benign forms with elevated HbA_2_ and HbF levels [16]. In recent years, it has emerged that KLF1 contributes to control adult and fetal globin gene expression. Accordingly, several reports have revealed that heterozygosity for loss-of-function mutations in KLF1 can ameliorate the severity of hemoglobinopathies by leading to persistent fetal globin gene expression in adult life (HPFH) [13,24,52,53,54]. Therefore, besides providing crucial information about the molecular processes that regulate the transcriptional regulation of the human fetal globin genes, these conditions have gained considerable clinical interest, given the potential therapeutic role of increased HbF levels in hemoglobinopathies. However, although the involvement of KLF1 in human erythropoiesis has been well-elucidated in recent years, there are still unresolved issues that require attention in order to fully understand the impact of various KLF1 mutations on these phenotypes.

Bearing this in mind and due to the paucity of functional studies on KLF1 mutations, we hence screened 17 subjects with β-thalassemia-like phenotypes for the presence of KLF1 gene variations with the aim of examining their possible contribution to globin gene expression. Nine individuals were found to be β-thalassemia carriers with a normal α-globin genotype while eight subjects had normal *HBB* and *HBA* genotypes with mild microcytosis and/or borderline or slightly increased levels of HbA_2_ and HbF. In these subjects, we identified thirteen KLF1 mutant alleles with seven sequence variants, including two yet-unreported mutations. Apart from two variants in the promoter region, the majority of these sequence variations were missense variants that fall in the transactivation and proline-rich domain. According to the classification introduced by Perkins et al., in 2016, such missense variations are class I variants with predicted minor functional consequences and a benign phenotype. Among them, the S102P variant was the most common KLF1 variation in the group of β-thalassemia carriers and was found either alone or in association with other sequence variations. The two novel variants were a nonsense (C94X) and a frameshift mutation (P173fxP236), respectively, both residing in the transactivation domain and causing aberrant amino acid terminations. According to Perkins et al., these variants are classified as class 3 mutations with expected mild hematological phenotypes [12].

In non-thalassemic individuals with KLF1 mutations, we noticed low or borderline MCV values associated with increased HbA_2_ levels. HbF levels in these patients ranged from normal to mildly elevated values. On the other hand, KLF1 mutations in combination with a β-thalassemia trait only resulted in further-increased HbA_2_ levels. These findings are consistent with previous reports [16,21,23] and led us to draw the conclusion that heterozygosity for these KLF1 variants has only modest or subtle effects on hematological parameters of β-thalassemia trait.

Notably, in some patients, we found combinations of different KLF1 sequence variations. In most cases, family studies allowed us to define the monoallelic or biallelic coinheritance of such KLF1 variants. Based on the evidence that amino acid modifications may be critically important for protein–protein interactions and could differently affect the transcriptional activity of KLF1, we asked whether these variant combinations could have additive or synergistic effects on KLF1 activity. To address this question, we performed functional analysis of wild-type and mutant KLF1 expression vectors in K562 cells. Unexpectedly, gene expression studies revealed that the S102P variant, so far reported as an SNP with non-pathogenic effects (Variant #0000566139 LOVD v.3.0) [47], is able to enhance the transcriptional activity of KLF1, thus acting as a gain-of-function mutation. Indeed, the S102P mutant construct was found associated with increased expression levels of genes that are direct targets of KLF1, such as β-globin, BCL11A, and ZBTB7A as compared to the wild-type construct, thus providing evidence that it is not a neutral polymorphism. Interestingly, this effect was even more evident when S102P was present in double mutants with either M39L or F182L that conversely, when present alone, exerted an opposite effect by slightly reducing KLF1 transcriptional activity. In these cases, we thus speculate that the combination of S102P with M39L or F182L may result in a higher degree of conformational alterations causing the enhanced transcriptional activity of KLF1. As regards to the effects on γ-globin gene expression, it is to be noted that, for most of the tested mutants, we did not find a significant inverse correlation with the expression levels of the transcriptional repressors of fetal globin genes, BCL11A and ZBTB7A, apart from the S102P + M39L double mutant. This can be explained by the evidence that the erythroleukemic K562 cell line, a common experimental tool used for the study of globin gene expression, shows an embryo-fetal globin gene expression pattern. Therefore, small changes in up-regulation of fetal globin gene expression may be obscured by the high background levels of γ-globin expression, thus making the interpretation of experimental results more difficult. On the other hand, as expected for class 3 mutations [12], constructs harboring C94X and P173fxP236 mutations are associated with a dramatic reduction of expression levels of KLF1 target genes and a slight increase in γ-globin transcripts. These findings are in line with previous studies showing reduced expression of BCL11A and ZBTB7A and increased levels of HBG1/2 in erythroid precursors with haploinsufficiency for KLF1 due to the presence of nonsense mutations [14].

Gene expression studies also allowed us to unveil the functional role of two known promoter variations, namely c. −251 (C > G) and c. −148 (G > A), reported as a neutral polymorphism (SNP) and pathogenetic variant, respectively [47,48]. Monoallelic inheritance of these two variants was detected in a subject (patient 16, Table 3) with mild microcytic anemia and slightly elevated HbF levels. This finding thus prompted us to investigate the effects of such combined variants on KLF1 expression. Consistently with literature data [16,23,47], gene reporter assays showed a significant reduction in the transcriptional activity of the c. −148 mutant promoter whereas, unexpectedly, the c. −251 single mutant construct induced a 40% increase in luciferase activity. Notably, the double mutant construct was able to partly reverse the inhibitory effects induced by c. −148 (G > A) alone, thus indicating that c. −251 (C > G) variation is not to be considered as a neutral SNP as it is partially dominant over the effects mediated by c. −148 (G > A) on the KLF1 promoter activity. 

Our study demonstrated the ameliorative effects on the thalassemia phenotype for some variants detected in our group of patients including c. −148 (G > A) and the two novel KLF1 haploinsufficiency mutations C94X and P173fxP236. More interestingly, in other cases so far referred to as neutral polymorphisms, such as the −251 (C > G) and S102P variants, our study also raised the notion that monoallelic inheritance of different variations may have unexpected additive effects leading to increased levels or enhanced transcriptional activity of KLF1, thus limiting or reversing the potential neutral or ameliorative effects of single mutations. Therefore, based on these results, our study adds new light to the significance of KLF1 mutations by showing that certain KLF1 variants may be causative of worsening effects on the thalassemia phenotype. Particularly in the case of SNPs or missense mutations of uncertain significance, functional studies are required to evaluate their significance and the possible cumulative effects of coexisting sequence variations that could differently contribute to KLF1 expression levels or transcriptional activity. 

## 5. Conclusions

Our study describes and provides functional characterization of C94X and P173fxP236, two novel KLF1 haploinsufficiency mutations causing increased fetal-globin gene expression. Notably, our study also highlights that some in cis combinations of KLF1 mutations may have worsening effects on the β-thalassemia phenotype. This study provides further insights into the multiple roles of KLF1 in erythropoiesis and highlights the idea that a subset of KLF1 mutations may contribute to the severity of the thalassemia phenotype, thus reinforcing the relevant implications of KLF1 screening for genetic counseling and for the effectiveness of prevention screening programs for hemoglobinopathies.

## Figures and Tables

**Table 1:** Primer sequences used for KLF1 amplification.

Primer Name	Primer	Sequence (5′ > 3′)	Amplicon Size (bp)
**KLF1 gene** (NG_013087.1)			
Exon 1	Forward	GATAGCAGCCTCCAACGTCTG	731 bp
	Reverse	CGTACCTCAGTCCTGGTTAAG	
Intron 1	Forward	CAGGGCCACAGTCACAGATC	885 bp
	Reverse	TCTTCGGAGCGCCACCACTG	
Exon 2	Forward	TCTGCGTCAGAGTGTCCAGC	768 bp
	Reverse	CACCTGGATCCTCTGCAGTC	
Exon 3	Forward	CAGTACCAAGGGCACTTCCAG	794 bp
	Reverse	CGTCCGTGTGAAGAGACCAC	
Exon 4	Forward	CTTGCACATGAAGCGCCACC	604 bp
	Reverse	TGTCACTGTAGTTTAGGAGATG	
Exon 5	Forward	GAAGGTCCCTTATTGTGGCTG	765 bp
	Reverse	CAGCCATTATTCTGGAGGTCA	
**KLF1 cDNA** (NM_006563.5)			
**EcoR1** KLF1 Forward		**GCGAATTCC**/ATGGCCACAGCCGAGACCGCCTTGCCCTC	1111 bp
**BglII** KLF1 Reverse		**GGAAGATCTTCC**GC/TCAAAGGTGGCGCTTCATGTGCAAGGC	
**KLF1promoter** (NG_013087.1)			
**XhoI** Prom KLF1 Forward		ATA**CTCGAG**/GATAGCAGCCTCCAACGTCTG	524 bp
**BglII** Prom KLF1 Reverse		GGA**AGATCT**TCC/TGGGCCTCAAGCCTCCTCTTC	
**HBBpromoter** (NG_059281.1)			
**Bgl II**-HBB Forward		GGAAGATCTTC/ACTCCTAAGCCAGTGCCAG	200 bp
**HindIII** HBB Reverse		CCCAAGCTTG/GGTTGCTAGTGAACACAGTTG	

**Table 2:** Primer sequences used for quantitative real-time PCR analysis.

Transcript	Accession Number	Primer	Sequence 5′-3′	Amplicon Size
HBB	NM_000518.5	Forward	ACACAACTGTGTTCACTAGCA	306 bp
		Reverse	ACTCAGTGTGGCAAAGGTG	
HBG1/HBG2	NM_000559.3	Forward	GACAGCTTTGGCAACCTGT	192 bp
	NM_000184.3	Reverse	CCAGGAGCTTGAAGTTCTC	
BCL11A	NM_022893.4	Forward	CGAGCACAAACGGAAACAATG	212 bp
		Reverse	CTCCAGTGCAGAAGTTTATCTG	
ZBTB7A	NM_015898.4	Forward	ACGAGTGCAACATCTGCAAG	128 bp
		Reverse	GGTCGTAGTTGTGGGCAAAG	
KLF1	NM_006563.5	Forward	CACACAGGGGAGAAGCCATA	149 bp
		Reverse	GTCAGAGCGCGAAAAAGC	
β2-microglobulin	NM_004048.4	Forward	CCGTGGCCTTAGCTGTGCT	150 bp
		Reverse	TCGGATGGATGAAACCGAGA	

**Table 3:** Hematological and genetic parameters of the subjects in examination.

Case	Age-Sex	Hb (g/dL)	MCV (fL)	HbF (%)	HbA_2_ (%)	HBB Genotype	KLF1 Genotype				
							Promoter		Coding Region		
							Mutation 1	Mutation 2	Mutation 1	Mutation 2	Mutation 3
1	13 y-M	12.2	52.2	1.1	6.1	β^0^39/N	--	--	M39L *	S102P *	--
2	20 y-F	8.1	72.2	4.6	5.3	β^0^IVSII-1/N	--	--	S102P	--	--
3	28 y-F	10.1	59.0	6.2	5.2	β^0^IVSI-1/N	--	--	S102P *	F182L *	--
4	28 y-F	8.9	64.8	1.2	5.1	β^0^39/N	−251 (C/G)	--	S102P	--	--
5	35 y-F	7.3	65.6	10.1	6.7	β^0^39/N	−251 (C/G)	−148 (G/A)	S102P	--	--
6	38 y-F	13.3	64.2	1.3	5.6	β^0^39/N	--	--	M39L *	S102P *	--
7	7 y-M	6.7	70.0	7.6	4.3	β^+^IVSI-110/N	--	--	--	--	--
8	15 y-M	11.6	57.0	3.3	4.8	β^0^IVSI-1/N	--	--	--	--	--
9	40 y-M	10.5	60.0	4.0	4.3	β^0^IVSII-1/N	--	--	--	--	--
10	9 y-F	13.2	73.3	1.8	3.7	N/N	--	--	C94X	--	--
11	13 y-F	12.5	75.6	1.9	3.5	N/N	--	--	C94X	--	--
12	30 y-M	14.9	90.0	0.8	3.2	N/N	−251 (C/G)	--	F182L	--	--
13	32 y-F	12.0	82.0	1.3	3.3	N/N	--	--	P173fsX236	--	--
14	34 y-F	11.8	79.0	2.0	3.6	N/N	--	--	C94X	--	--
15	56 y-M	14.5	83.0	6.6	2.7	N/N	--	--	S102P *	F182L *	P173fsX236
16	73 y-M	10.7	70.0	3.6	2.8	N/N	−251 (C/G) *	−148 (G/A) *	P173fsX236	--	--
17	30 y-F	15.5	88.3	0.5	3.5	N/N	--	--	--	--	--

M: male; F: female; *: in cis mutations.

## Data Availability

No new data were created or analyzed in this study. Data sharing is not applicable to this article.

## Supplementary Materials

The following supporting information can be downloaded at: https://www.mdpi.com/article/10.3390/biology12040510/s1, Figure S1: Original image of western blot analysis shown in Figure 4.
