# Supplementary figures and images for: Identification and Functional Analysis of Known and New Mutations in the Transcription Factor KLF1 Linked with β-Thalassemia-like Phenotypes
Source: Biology (Basel). 2023 Mar 28;12(4):510. doi: 10.3390/biology12040510 (PMC10135830; doi:10.3390/biology12040510)

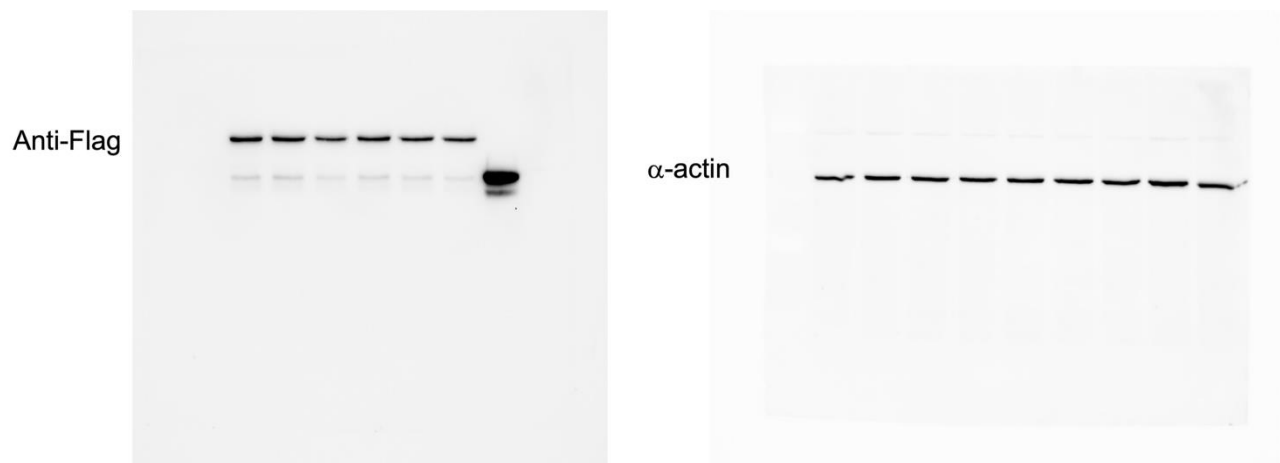

**Figure S1.** Original image of western blot analysis shown in Figure 4.

Supplement: Supplementary file 1 [file biology-12-00510-s001.zip › biology-2249790-supplementary.pdf]
